# Cross-cultural adaptation and validation of a Norwegian version of the Goodman Satisfaction Score (GSS-NO) for patients with total hip and knee arthroplasty

**DOI:** 10.2340/17453674.2024.42703

**Published:** 2025-01-10

**Authors:** Ingvild Buset BERGVAD, Anders KOTTORP, Arild AAMODT, Anners LERDAL, Søren T SKOU, Maren Falch LINDBERG

**Affiliations:** 1 Department of Surgery, Lovisenberg Diaconal Hospital, Oslo, Norway; 2 Department of Interdisciplinary Health Sciences, Institute of Health and Society, Faculty of Medicine, University of Oslo, Norway; 3 Faculty of Health and Society, Malmö University, Malmö, Sweden; 4 The Research and Implementation Unit PROgrez, Department of Physiotherapy and Occupational Therapy, Næstved-Slagelse-Ringsted Hospitals, Slagelse, Denmark; 5 Research Unit for Musculoskeletal Function and Physiotherapy, Department of Sports Science and Clinical Biomechanics, University of Southern Denmark, Odense, Denmark; 6 Department of Public Health Science, Institute of Health and Society, Faculty of Medicine, University of Oslo, Oslo, Norway

## Abstract

**Background and purpose:**

Measuring patient satisfaction after total hip (THA) and total knee arthroplasty (TKA) is important. We aimed to cross-culturally adapt and examine the psychometric properties of the self-reported Goodman Satisfaction Score (GSS) in a sample of Norwegian patients following primary THA and TKA.

**Methods:**

The GSS was translated and adapted into Norwegian (GSS-NO) following standard guidelines. 800 patients from the Norwegian Arthroplasty Register who had undergone surgery 6–11 months prior were invited to complete GSS-NO and questions on sociodemographic factors, pain, and function in a cross-sectional study. We examined validity in relation to internal structure, response processes, and precision using Rasch analysis, relationships between the GSS-NO and pain and function using Pearson’s correlation coefficients, and test–retest reliability using linear weighted kappa statistics.

**Results:**

The GSS-NO was adapted with few challenges. 404 patients (49% THA, 51% TKA) returned complete answers. The GSS-NO met all criteria regarding the rating scale functioning. Local independence among items and unidimensionality was supported and there was acceptable goodness-of-fit. The internal consistency was 0.94. We found no systematic differential item functioning by age, sex, work status, education, cohabitation status, or hip or knee surgery. The correlation coefficients between GSS-NO and pain and function outcomes were 0.79 (95% confidence interval [CI] 0.76–0.82) and 0.79 (CI 0.76–0.82), respectively. Test–retest reliability with weighted kappa ranged from 0.43–0.55 for THA and 0.54–0.81 for TKA.

**Conclusion:**

The cross-cultural adaptation of GSS-NO proved to be a valid and reliable measure for use in Norwegian-speaking patients following primary THA and TKA.

Accurately assessing patient outcomes of total hip arthroplasty (THA) and total knee arthroplasty (TKA) through patient-reported outcome measures (PROMs) is essential to optimize patient outcomes. Satisfaction with one’s surgical outcome does not necessarily correlate with measures of treatment effectiveness such as pain reduction and functional improvements [1]. Thus, specifically measuring patients’ satisfaction with their surgical outcome allows for a more comprehensive understanding, considering subjective factors like expectations and preferences [1].

Although numerous studies report satisfaction following primary THA and TKA, a variety of methods are used, and few studies use validated satisfaction instruments [2]. In 2020, Goodman et al. proposed a new self-reported measure of satisfaction with THA and TKA outcome, the Goodman Satisfaction Score (GSS) [3]. The measure consists of 4 items assessing patient satisfaction and 1 item assessing quality of life following primary THA and TKA, thus allowing for satisfaction to be measured in several dimensions that matter to the patients. Initial evaluation of the measure showed that it is feasible, valid, and reliable with high internal consistency [3].

The use of a measure in a country, language or culture other than the one it was developed for requires a thorough process to ensure that its content validity is maintained. This process not only includes linguistic translation, but also cultural adaptation of each item, the response alternatives, and the instructions for the measure [4]. The GSS has been adapted and validated for use in Spanish (THA) [5] and Italian (THA/TKA) [6], with psychometric properties comparable to the original measure.

We aimed to translate the GSS into Norwegian, culturally adapt it, and examine its psychometric properties in a population of Norwegian patients following primary THA and TKA.

## Methods

### Design

The GSS was translated and adapted to Norwegian according to the guidelines described by Beaton et al. [4], and its psychometric properties were assessed in a cross-sectional survey design. The study is reported guided by the COSMIN reporting guideline for studies on measurement properties of patient-reported outcome measures [7].

### Cross-cultural adaptation

The process of cross-cultural adaptation consisted of translation, back translation, and pretesting with interviews [4] as described in Table 1. Patients (n = 5) were recruited for the pretesting interviews after TKA surgery at Lovisenberg Diaconal Hospital, after agreeing to be contacted by researchers. Before participating in the interview by phone, patients signed written consent forms.

**Table 1:** Overview of the translation and cultural adaptation process [4]

Stages	Description
I	Forward translation 2 separate translations from English to Norwegian Both translators were native Norwegian speakers and had excellent English skills and knowledge of the patient population
II	Synthesis In addition to the translators, a native English-speaking medical doctor with excellent Norwegian skills, was invited to provide insight into the translations Discrepancies requiring discussion were: - The response option “More improvement than I ever dreamed possible” as the use of “dreamed” is culturally unsuitable in Norwegian. It was changed to: “More improvement than I could have imagined” - The use of “little” and “very” instead of “some” and “huge” All disputes were resolved and 1 common translation was produced
III	Back translation 2 separate back translations from Norwegian to English 1 translator within the medical field, and 1 translator with no medical background Both back translations were sent to the corresponding author of the original GSS, who provided feedback
IV	Expert committee review The committee consisted of the 4 translators, 2 senior researchers, and a clinician working with THA and TKA patients All components of the score were reviewed A prefinal version of the Norwegian version was created
V	Pretesting (interviews) 5 patients who had undergone TKA during the previous year completed the score and were interviewed by telephone The subjects were probed regarding their understanding of the questions and the response alternatives Saturation of information was reached after 5 interviews The Norwegian version was found to be face valid A final Norwegian version was completed

### Patients and data collection

A sample of 800 patients over the age of 18 who had undergone either THA (n = 400) or TKA (n = 400) during the last 6–11 months were randomly selected from the Norwegian Arthroplasty Register. The register reports inclusion of 97% of all THA/TKA in Norway [8]. We approached patients at between 6 and 11 months postoperatively in an attempt to minimize ceiling effect, expecting that patients at this time point could provide greater variance in satisfaction scores.

The patients received questionnaires and a written informed consent form by mail. Those who wished to participate signed the consent form, filled in the questionnaires, and returned them in a sealed prepaid envelope. Data collection was carried out between May and August 2022. Test–retest reliability was assessed by sending the GSS-NO a second time immediately as each patient’s initial completed questionnaire was received, because we had to allow up to 14 days for the mail to reach patients. Reminders were not sent.

### Measurements

#### Sociodemographic variables

Sociodemographic variables included sex, age, work situation, level of education, and cohabitation. The variables were dichotomized; age (< 70 years, ≥ 70 years), work situation (in paid work, not in paid work: including both patients of working age and retired patients), level of education (low < 13 years of education, high ≥ 13 years), cohabitation (living alone, living with others or in institution).

#### Goodman Satisfaction Score

The GSS consists of 4 questions assessing patients’ satisfaction with the result following THA and TKA in relation to pain relief, ability to do house and yard work, ability to do recreational activities, and overall satisfaction, and 1 question assessing how much surgery has improved their quality of life (QoL). The satisfaction questions are rated on a 5-step Likert scale, ranging from “very satisfied,” “somewhat satisfied,” “neither satisfied nor dissatisfied,” “somewhat dissatisfied,” to “very dissatisfied,” with the scale steps being assigned scores of 100, 75, 50, 25, and 0, respectively. Total satisfaction scores are calculated as the mean of the 4 satisfaction item scores, with higher scores indicating greater satisfaction. A total satisfaction score was not calculated if any component was missing. The QoL question is rated on a 6-step Likert scale ranging from “more improvement than I ever dreamed possible” to “the quality of my life is worse” (“dreamed” was changed to “imagined” in the Norwegian version during the cross-cultural adaptation) [3]. This question is rated from 1–6, with higher scores corresponding to worse outcomes. The QoL item is not included in the total satisfaction score.

#### Pain and function

Pain and function were measured using the Western Ontario and McMaster Universities Osteoarthritis Index (WOMAC) scores [9], as calculated from the Hip Osteoarthritis Outcome Score (HOOS) for THA patients and the Knee injury and Osteoarthritis Outcome Score (KOOS) for TKA patients. The HOOS and KOOS are both extensions of the WOMAC [10]. The separate subscales for WOMAC pain (derived from 5 items) and WOMAC function (derived from 17 items) were transformed to 0–100 scales, where higher scores indicate worse outcomes. Calculating WOMAC scores allowed for hip and knee patients to be analyzed together.

### Statistics

Validity in relation to internal structure, response processes, and precision was evaluated using a 2-faceted Rasch rating scale model [11] and the Winsteps Rasch measurement computer program version 5.2.3 [12]. SPSS version 28 (IBM Corp, Armonk, NY, USA) was used for descriptive statistics, summarizing GSS-NO scores, validity in relation to other variables (Pearson’s correlation coefficients), and for evaluating test–retest reliability (weighted kappa statistics). We structured our analysis based on the proposed aspects of validity from the Standards of Education and Psychological Testing (2014) [13].

#### Rasch analysis

As the fifth item of the GSS represents a different construct (QoL), we decided to focus on the 4 satisfaction items.

The Rasch analysis was performed using the following steps:

- In Step 1, the rating scale functioning of the 5-category rating scale was investigated to determine whether (a) more than 10 responses for each rating scale category were achieved, (b) the average measures on each item for each category advanced monotonically, and (c) these were associated with outfit mean square (MnSq) values of less than 2.0 for each of the category calibrations [16].
- Step 2 included 3 parts:
  2a The Rasch model’s assumption of local independence among the GSS-NO was explored by monitoring the standardized correlations between the item score residuals [14]. A criterion of shared variance between item score residuals no higher than 50% (corresponding to a correlation coefficient no higher than 0.7) was used to support local independence among items [15].
  2b The goodness-of-fit of each of the GSS-NO satisfaction questions [11] was also evaluated. Acceptable item goodness-of-fit was defined as Infit MnSq values between 0.7 and 1.3, which is stricter than the suggested guidelines for surveys [16].
  2c The unidimensionality of the GSS-NO satisfaction questions was evaluated by a principal component analysis (PCA) of the residuals, with the criterion that the first latent dimension should explain at least 50% of total variance, in line with earlier studies [17,18]. The eigenvalue of the secondary dimension (reported as first contrast) was also monitored, using a cut-off of 2.0 or higher to signal a lack of convergence in the data.
- Step 3 evaluated aspects of person response validity. The criterion for evaluating person goodness-of-fit was to reject Infit MnSq values of 1.4 logits or higher associated with a z-value of 2 or higher, accepting that by chance 5% of the sample may fail to demonstrate acceptable goodness-of-fit without threatening evidence of person response validity [16,19]. Evidence of any floor or ceiling effects in the responses of the GSS-NO was also monitored, and the targeting of the GSS-NO to the respondents was assessed using the Wright map output from the Winsteps program [12].
- In Step 4, the person-separation reliability index was calculated to determine whether the GSS-NO could distinguish respondents demonstrating different levels of perceived satisfaction. A criterion that the GSS-NO should be able to distinguish at least 3 groups (indicating high, medium, and low levels of perceived satisfaction) was used and requires a person separation index of at least 2.0 [20]. Internal consistency was also assessed using the Kuder–Richardson Formula 20 [21], equivalent to Cronbach’s alpha.
- Lastly, in Step 5, several differential item functioning (DIF) analyses were conducted to investigate whether subgroups in the sample had significantly different response patterns from items despite equal levels of the underlying trait. DIF was evaluated across the following subgroups: age, sex, work, education, cohabitation, hip or knee surgery. DIF was analyzed using the Mantel–Haenzel statistical approach for polytomous data with a Bonferroni adjusted P value < 0.01 [22].

#### Relationship to other variables

In addition to the Rasch analysis, Pearson’s correlation coefficients were used to evaluate the relationships between GSS-NO and WOMAC pain and function outcomes. We hypothesized that, like the English version, the Norwegian version would correlate moderately with the WOMAC pain and function subscales. The relationship between the Rasch-generated satisfaction measure and the total sum score of the 4 satisfaction items was also calculated to determine whether the satisfaction sum score could be used as an interval estimate. Correlation coefficients > 0.9 were interpreted as very high, 0.7–0.9 as high, 0.5–0.7 as moderate, 0.3–0.5 as low, and < 0.3 as negligible [23].

#### Test–retest reliability/precision

The second (retest) questionnaire included an anchor question: “Have you had any new problems involving the joint(s) in question since you answered our first questionnaire?” Patients responding “yes” to this question were excluded from the analysis of test–-retest reliability. Linear weighted kappa statistics were then calculated to assess test–retest reliability. Kappa scores were interpreted using Altman’s guidelines, with < 0.20 considered “poor” reliability, 0.21–0.40 “fair,” 0.41–0.60 “moderate,” 0.61–0.80 “good,” and 0.81–1.0 “very good,” as shown in Table 4 [24].

### Ethics, data sharing, use of AI, funding, and disclosures

The study was approved by the Regional Committee for Medical Ethics, Southeastern Norway (#2017/968). No AI was used. The authors have no conflict of interests to declare. Complete disclosure of interest forms according to ICMJE are available on the article page, doi: 10.2340/17453674.2024.42703

## Results

### Cross-cultural adaptation

During the translation process, 1 response item (“More improvement than I ever dreamed possible….”) required discussion before consensus was reached, and “ever dreamed possible” was changed to “could have imagined.” The GSS-NO was found to be face valid in the interviews with the 5 patients who had undergone TKA, and only minor changes in wording were necessary. Saturation of information was considered achieved as patients 4 and 5 did not contribute new information.

### Patients/demographics

404 patients returned complete answers (49% THA, 51% TKA). Mean age was 70 years, 64% were female, 40% had higher education, and 25% lived alone (Table 2). Patient satisfaction levels were high (Table 3), with 93% of THA and 87% of TKA being very or somewhat satisfied with their overall surgical results. The proportion of patients answering “very satisfied” was higher in THA than TKA on all 4 satisfaction questions.

**Table 2:** Patient demographic characteristics. Values are count (%) unless otherwise specified

Demographic characteristics	Overall (n = 403)	THA (n = 196)	TKA (n = 207)
Age, mean (SD)	70.5 (9.1)	70.1 (9.5)	70.2 (8.8)
Sex			
Female	261 (65)	133 (68)	128 (62)
Male	142 (35)	63 (32)	79 (38)
Work status			
In paid work	76 (19)	41 (21)	35 (17)
Not in paid work	322 (80)	154 (79)	168 (83)
Missing data	5	1	4
Educational status			
Lower education	243 (61)	113 (58)	130 (63)
Higher education	157 (39)	81 (42)	76 (7)
Missing data	4	2	1
Cohabitation status			
Living alone	97 (24)	53 (27)	44 (21)
Living with others or			
in institution	306 (76)	143 (73)	163 (79)

SD = standard deviation; THA = total hip arthroplasty; TKA = total knee arthroplasty.

**Table 3:** Goodman Satisfaction Score response distributions by arthroplasty type. Values are count (%)

Questions and responses	THA (n = 196)	TKA (n = 207)
Pain relief		
Very satisfied	167 (86)	147 (71)
Somewhat satisfied	16 (8.2)	30 (15)
Neither satisfied nor dissatisfied	5 (2.6)	7 (3.4)
Somewhat dissatisfied	5 (2.6)	14 (6.8)
Very dissatisfied	2 (1.0)	8 (3.9)
Missing data	1	1
Improving ability to do housework or yard work		
Very satisfied	140 (72)	116 (56)
Somewhat satisfied	25 (18)	47 (23)
Neither satisfied nor dissatisfied	11 (5.6)	16 (7.8)
Somewhat dissatisfied	7 (3.6)	20 (9.7)
Very dissatisfied	2 (1.0)	7 (3.4)
Missing data	1	1
Improving ability to do recreational activities		
Very satisfied	114 (59)	88 (43)
Somewhat satisfied	51 (26)	67 (33)
Neither satisfied nor dissatisfied	17 (8.8)	19 (9.2)
Somewhat dissatisfied	8 (4.1)	19 (9.2)
Very dissatisfied	4 (2.1)	13 (6.3)
Missing data	2	1
Overall satisfaction with surgery results		
Very satisfied	161 (83)	148 (72)
Somewhat satisfied	20 (10)	31 (15)
Neither satisfied nor dissatisfied	5 (2.6)	5 (2.4)
Somewhat dissatisfied	6 (3.1)	10 (4.9)
Very dissatisfied	2 (1.0)	12 (5.8)
Missing data	2	1
How much did your hip/knee surgery improve your QoL?		
More improvement than I ever dreamed possible	74 (38)	60 (29)
Great improvement	99 (51)	95 (46)
Moderate improvement	18 (9.2)	26 (13)
A little improvement	1 (0.5)	12 (5.8)
No improvement	2 (1.0)	11 (5.3)
The quality of my life is worse	1 (0.5)	3 (1.4)
Missing data	1	0

QoL = quality of life; THA = total hip arthroplasty; TKA = total knee arthroplasty.

### Rasch analysis

In Step 1, the rating scale met all set criteria, as shown in Figure. When monitoring the rating scale functioning, rating scale category 3 (Neither satisfied nor dissatisfied) had a lower probability of being used than expected, but it still met the criteria for acceptable functioning.

**Figure:**
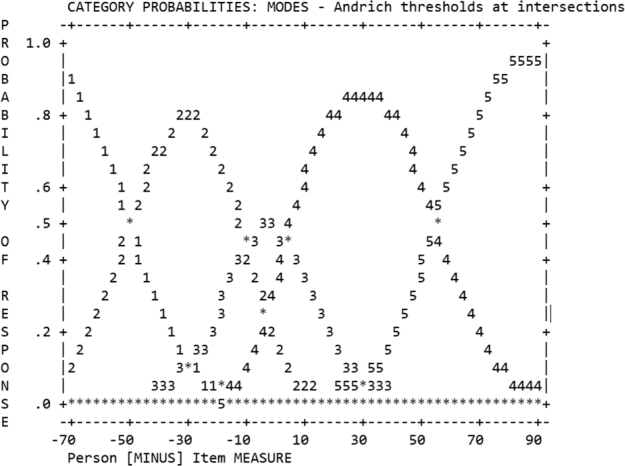
Andrich thresholds at intersections for use of the rating scale, showing that each of the rating scale categories had distinct higher probability of being used in a logical order along the continuum of person–item difference.

In Step 2a, assessment of the standardized correlations between the item score residuals indicated that the largest shared variance between item score residuals was between items #1 (pain) and #2 (house or yard work) with a correlation coefficient of r = 0.26, representing a shared variance of less than 7.0%. Thus, we concluded that local independence among the items was supported.

In Step 2b, all 4 items met our set criterion for acceptable goodness-of-fit, with a range of fit statistics between 0.85 and 1.09.

Step 2c supported the unidimensionality of the scale, as the first latent dimension explained 80% of total variance, and the eigenvalue of the secondary dimension was 2.0.

In Step 3, 13 person records in our sample (2.2%) failed to demonstrate acceptable goodness-of-fit. 7 person records demonstrated minimum scores (1.7%), and 196 person records demonstrated maximum scores (48.6%). Targeting of the GSS-NO to the respondent scores demonstrated a clear mismatch.

Step 4 showed that the GSS-NO could distinguish respondents in 3 distinct levels/groups of perceived satisfaction, with a person separation index of 2.00 (including both extreme and non-extreme responses). Internal consistency was 0.94.

In Step 5, no significant DIF was found across any of the subgroups: age, sex, work, education, cohabitation, and hip or knee surgery. Thus, we concluded that the GSS-NO did not demonstrate any systematic significant bias in relation to sociodemographic variables.

### Relationship to other variables

The correlation coefficients between the total satisfaction scores and the WOMAC pain and function outcomes were 0.79 (95% confidence interval [CI] 0.76–0.82] and 0.79 (CI 0.76–0.82), respectively. In addition, the correlation coefficient between the total satisfaction scores and the Rasch-generated measures was 0.98 (CI 0.98–0.99) indicating that total satisfaction score could be used as a valid approximate of patients’ overall satisfaction.

### Test-retest reliability/precision

The retest questionnaire was continuously mailed to patients until we had a minimum of 50 answers. It was mailed to 228 patients, and 208 (91%) were returned. Of these, 22 were excluded because they had answered “yes” to the question “Have you had any new problems involving the joint(s) in question since you answered our first questionnaire?” and 2 were excluded because of missing answers. 184 patients (81%), 92 THA and 92 TKA patients, were included in the test–retest reliability analysis. Mean time between completion of the test and retest was 15.3 days, range 5–41 days (median 14). The kappa coefficients and their interpretations are presented in Table 4. All questions had moderate to very good reliability (linear weighted kappa range 0.43–0.81) according to Altman’s guidelines for interpretation [24].

**Table 4:** Linear weighted kappa coefficients and their interpretations

Outcome	Weighted kappa (CI)	Standard error	P value	Interpretation
THA+TKA				
Pain	0.68 (0.58–0.79)	0.06	< 0.001	Good
Work	0.53 (0.43–0.64)	0.06	< 0.001	Moderate
Rec.	0.64 (0.55–0.73)	0.05	< 0.001	Good
Overall	0.73 (0.62–0.83)	0.05	< 0.001	Good
QoL	0.64 (0.56–0.73)	0.04	< 0.001	Good
THA				
Pain	0.55 (0.34–0.75)	0.10	< 0.001	Good
Work	0.49 (0.31–0.66)	0.09	< 0.001	Moderate
Rec.	0.43 (0.26–0.59)	0.08	< 0.001	Moderate
Overall	0.52 (0.31–0.73)	0.11	< 0.001	Good
QoL	0.51 (0.35–0.67)	0.08	< 0.001	Moderate
TKA				
Pain	0.73 (0.61–0.84)	0.06	< 0.001	Good
Work	0.54 (0.40–0.68)	0.07	< 0.001	Moderate
Rec	0.74 (0.63–0.84)	0.05	< 0.001	Good
Overall	0.81 (0.70–0.91)	0.06	< 0.001	Very good
QoL	0.71 (0.61–0.81)	0.05	< 0.001	Good

Label	Question			

Pain	(a) For relieving pain?			
Work	(b) For improving your ability to do housework or yard work?			
Rec.	(c) For improving your ability to do recreational activities?			
Overall	(d) Overall, how satisfied are you with the results of your hip/knee surgery?			
QoL	(e) How much did your hip or knee surgery improve the quality of your life?			

Reliability determined using Altman’s guidelines for interpretation [24]				
≤ 0.20	Poor			
0.21–0.40	Fair			
0.41–0.60	Moderate			
0.61–0.80	Good			
0.81–1.00	Very good			

CI = 95% confidence interval; Also see Table 3.

## Discussion

In this study, we describe the cross-cultural adaptation and validation of the Norwegian version of the GSS. Our results indicate that the adapted GSS-NO is feasible and has psychometric properties consistent with the original version. Thus, we present a brief and simple instrument that is a valid and reliable option, with low response burden, to assess satisfaction following primary THA and TKA in a Norwegian population.

The patients in our study reported high levels of satisfaction with 86% of THA and 71% of TKA patients answering “very satisfied” when asked to rate their overall satisfaction with their surgical outcome. This is comparable to the North American sample in the original article where 91% of THA and 78% of TKA patients reported being very satisfied overall [3]. For the Spanish version evaluated among South American THA patients, 75% reported being very satisfied overall [5]. The Italian study did not report on satisfaction levels [6].

We observed a strong ceiling effect in our sample, which from a clinical perspective is good (i.e., high levels of satisfaction), but from a psychometric perspective is more problematic. This is in accordance with findings from both the original article [3] and the Italian version [6]. It is well known that most patients are satisfied following primary THA and TKA [25]; a ceiling effect of satisfaction is therefore to be expected. No floor effect was observed, as only 1.5% of patients obtained the lowest possible score. Maximum and minimum scores do not contribute to the psychometric evaluation of items and/or persons within the Rasch model used, thus our large sample of over 400 patients was justified, as we expected a ceiling effect.

Interestingly, our study showed high correlations between the GSS-NO and WOMAC pain and function subscales. The correlations were higher than in the original article where correlations were moderate [3]. The Spanish version of the GSS was also found to correlate moderately with pain and function measured with the Oxford Hip Score [5] and the Italian GSS had moderate correlations with the HOOS/KOOS [6]. One can speculate as to why our findings were so different. The Norwegian sample may differ from the North American, South American (Chile), and Italian samples in numerous ways, such as access to healthcare, economic concerns regarding healthcare, expectations before surgery – as this is known to influence satisfaction [1], not to mention differences in the questionnaires used (WOMAC vs Oxford Hip Score). It would be interesting to compare these results with those in other Scandinavian countries, where the culture and healthcare systems are more similar, using the same questionnaire.

The Rasch analysis found that rating scale category 3 (neither satisfied nor dissatisfied) had a lower probability of being used as a response than expected, but still met the set criteria for retention in the scale. Although this could indicate a psychometric weakness, we do not consider it to be a problem in our data as patients seem to view their surgical outcome as either satisfactory or unsatisfactory.

Although satisfaction can be influenced by a number of factors varying between patients and thus be challenging to measure [26], satisfaction with outcome of surgery is still important to measure as part of patient-centered care and to better understand the clinical results. Satisfaction with the outcome is better measured using specifically designed questionnaires like the GSS-NO rather than questionnaires measuring well-known influencers of satisfaction, mainly pain and function, or single-item measures that are not capable of capturing the complexity and depth of satisfaction [1]. Although caution is needed when interpreting the results, we cannot simply avoid or minimize measuring patients’ satisfaction with the outcome following THA and TKA because it is a difficult concept to grasp.

### Strengths

Our adaptation of the GSS into Norwegian followed the strict guidelines outlined by Beaton et al. [4]. The number of participants in this study, and the fact that they were invited from all over Norway, supports the generalizability of our results. The response rate of 51%, without reminders, is an acceptable response rate. Although reminders may have increased the response rates, we found no significant differences between responders and non-responders with regard to age, sex, and whether they had undergone THA or TKA, suggesting that our sample was representative of the patient population with respect to these characteristics. Moreover, our main aim was to evaluate the GSS-NO’s psychometric properties, not the actual results in the population and, thus, the 51% response rate is unlikely to have impacted our findings. Our use of Rasch analysis is also a novel approach that adds to previous validation studies of this instrument.

### Limitations

Although the number of patients who were interviewed and probed concerning the GSS after its translation were far fewer (n = 5) than recommended, information saturation was reached and all 5 of the interview subjects provided similar feedback, suggesting that additional interviews were unlikely to yield new information. As we tested the GSS-NO only in patients following primary THA and TKA, our findings may not be generalizable to patients who have undergone other types of arthroplasty (such as revision surgery or unicompartmental knee arthroplasty). For the purpose of the DIF analysis, the sociodemographic variables were dichotomized. While we aimed to select meaningful cut-points, it is possible that the DIF results could differ had other cut-points been chosen.

### Conclusion

We successfully adapted and validated a Norwegian version of the GSS for the assessment of satisfaction following primary THA and TKA. The GSS-NO could fill the need for a validated, feasible measure of satisfaction with low respondent burden, which measures several aspects of patients’ satisfaction with the outcome following THA and TKA. It is suitable not only for research purposes, but also to monitor and improve clinical practice.

Future research investigating the relationships between satisfaction, expectations, cultural settings etc., would be helpful to gain more knowledge of the difficult concept of satisfaction. Our study supports the adaptation of the GSS to further languages.

## Supplementary Material



## Notes

IBB, AA, AL, STS, and ML conceived and designed the study. IBB, AL, and ML performed the data acquisition. AK and IBB performed the statistical analyses. IBB drafted the manuscript. All the authors contributed to interpretation of the findings, preparation and critically revisions of the manuscript, and have read and approved the final manuscript before submission.

The authors would like to thank Caryl Gay for assistance performing the weighted kappa statistics and providing language editing.

Handling co-editor: Taco Gosens

*Acta* thanks David N Bernstein and Siri Heijbel for help with peer review of this manuscript.
