# Supplementary material for: Cross-cultural adaptation and validation of a Norwegian version of the Goodman Satisfaction Score (GSS-NO) for patients with total hip and knee arthroplasty
Source: Acta Orthop. 2025 Jan 10;96:52–8. doi: 10.2340/17453674.2024.42703 (PMC11724478; doi:10.2340/17453674.2024.42703)
Supplement: Supplementary file 1 [file ActaO-96-42703-s1.pdf]

Fornøydhet og livskvalitet etter hofte- og/eller kneproteseoperasjoner

1. Spørsmål om fornøydhet

Hvor fornøyd er du med resultatet av proteseoperasjonen på følgende områder?
Vennligst sett ett kryss for hvert spørsmål. Dersom/hvis du har fått proteser i begge hofter eller knær, svar/angi hvor generelt fornøyd/misfornøyd du er.

	Veldig fornøyd	Litt fornøyd	Verken fornøyd eller misfornøyd	Litt misfornøyd	Veldig misfornøyd
Med hensyn til bedring av smerter?	☐	☐	☐	☐	☐
Med hensyn til bedring av din evne til å jobbe i hus eller hage?	☐	☐	☐	☐	☐
Med hensyn til bedring av din evne til å gjennomføre fritidsaktiviteter?	☐	☐	☐	☐	☐
Totalt sett, hvor fornøyd er du med resultatet av proteseoperasjonen?	☐	☐	☐	☐	☐

2. Spørsmål om livskvalitet

Hvor mye har proteseoperasjonen bedret din livskvalitet?

Større bedring enn jeg kunne forestilt meg	Stor bedring	Moderat bedring	En liten bedring	Ingen bedring	Min livskvalitet har blitt bedre
☐	☐	☐	☐	☐	☐
